# Cost‐saving population genomic investigation of *Daphnia longispina* complex resting eggs using whole‐genome amplification and pre‐sequencing screening

**DOI:** 10.1002/ece3.9682

**Published:** 2022-12-27

**Authors:** Jana Nickel, Mathilde Cordellier

**Affiliations:** ^1^ Institute of Animal Cell and Systems Biology University of Hamburg Hamburg Germany

**Keywords:** *Daphnia longispina* species complex, population genomics, resting egg bank, whole‐genome amplification

## Abstract

Resting stages of aquatic organisms that accumulate in the sediment over time are an exceptional resource that allows direct insights into past populations and addressing evolutionary questions. This is of particular interest in taxa that face relatively new environmental challenges, e.g., climate change and eutrophication, such as the *Daphnia longispina* species complex, a keystone zooplankton group in European freshwater ecosystems. However, genomic analysis might be challenging as DNA yield from many of these resting stages can be low and the material degraded. To reliably allow the resequencing of single *Daphnia* resting eggs from different sediment layers and characterize genomic changes through time, we performed whole‐genome amplification to obtain DNA amounts suitable for genome resequencing and tested multiple protocols involving egg isolation, whole‐genome amplification kits, and library preparation. A pre‐sequencing contamination screening was developed, consisting of amplifying mitochondrial *Daphnia* and bacterial markers, to quickly assess and exclude possibly contaminated samples. In total, we successfully amplified and sequenced nine genomes from *Daphnia* resting eggs that could be identified as *Daphnia longispina* species. We analyzed the genome coverage and heterozygosity of these samples to optimize this method for future projects involving population genomic investigation of the resting egg bank.

## Graphical abstract

Resting stages of aquatic organisms that accumulate in sediment can give us direct insights into past populations and environmental changes. However, researchers often face challenges with the genomic investigation of resting stages because of the low DNA quality and quantity. We developed a method for the reliable resequencing of single *Daphnia longispina* complex resting eggs using whole‐genome amplification and a pre‐sequencing contamination screening and tested whether they are suitable for further population genomic analysis.

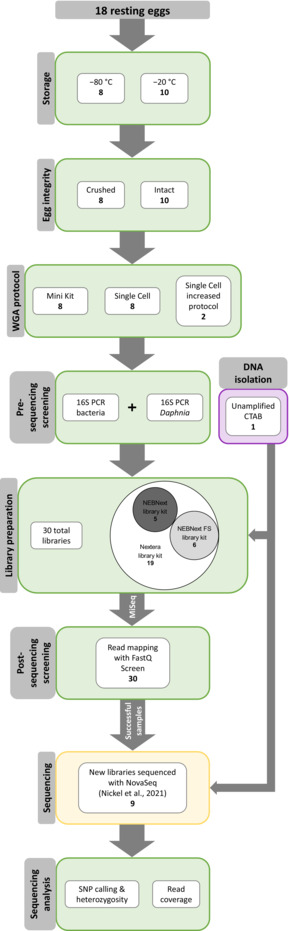

## 1 INTRODUCTION

Organisms that produce dormant propagules, e.g., seeds, eggs, cysts, or spores are especially interesting to investigate the evolutionary history of species, populations, and whole ecosystems. These dormant propagules accumulate in layers of limnic or marine sediment over time and function as a biological archive that allows direct insights into shifts in genetic variation and how past and ongoing environmental changes have shaped ecosystems (Hairston, 1996; Orsini et al., 2016). Combined with the rapidly growing genomic resources and high‐throughput sequencing technologies, resting stage banks enabled researchers to investigate local adaptation in rotifers (Franch‐Gras et al., 2018), genetic structure in diatoms (Aloisi et al., 2011), and genetic diversity over time in *Daphnia magna* (Orsini et al., 2016). In freshwater sediment, resting egg banks are often dominated by zooplanktonic crustaceans such as the genus *Daphnia* (Crustacea: Cladocera) that play a key role in aquatic food webs; they graze on phytoplankton and are a food source for secondary consumers (Lampert & Sommer, 2007). As cyclical parthenogens, *Daphnia* are able to switch between asexual and sexual reproduction and the resulting resting eggs can withstand adverse conditions for decades and even centuries (Frisch et al., 2014). In some cases, resting eggs extracted from sediment cores can be hatched and clonal lines brought back to life to investigate temporal and spatial patterns in the recent past (Orsini et al., 2013). The DNA preserved in those resting eggs can also be directly analyzed with various molecular methods (Cousyn et al., 2001; Lack et al., 2018; Limburg & Weider, 2002) to study adaptation to changing environmental conditions such as temperature (Dziuba et al., 2020) or eutrophication (Alric et al., 2016; Cordellier et al., 2021).

Genomic investigation of the resting egg bank that can be conducted directly without hatching and establishing clonal lines is hindered by small amounts of potentially degraded DNA in single *Daphnia* resting eggs. Before high‐throughput sequencing technologies became widespread in non‐model organisms, population genetic studies were restricted to a few nuclear and mitochondrial markers to characterize resting eggs (Brede et al., 2009; Möst et al., 2015; Ortells et al., 2014). However, the small amount of tissue (~3500 cells per diapausing embryo in *D. magna*; Chen et al., 2018) makes whole‐genome sequencing extremely challenging. One possible approach is pooling multiple eggs from a population for whole‐genome sequencing but then information on individual genotypes is lost (Cordellier et al., 2021).

Another approach to obtain sufficient DNA from starting material that is of limited quantity and/or quality is multiple displacement amplification (MDA), a method for whole‐genome amplification (WGA) commonly used to perform isothermal amplification of the template DNA. MDA‐WGA uses phi29 DNA polymerase and annealing of random hexamers which does not require species‐specific primers and yields an average DNA product length of >10 kb (Dean et al., 2002; Spits et al., 2006). It is the preferred method for SNP detection (de Bourcy et al., 2014) and has been used in other studies where extremely small specimens hinder genomic investigation to successfully perform RADSeq (Cruaud et al., 2018) and whole‐genome sequencing (O'Grady et al., 2021). These new methods enabled researchers to study introgression in Schistosome parasites (Platt II et al., 2019) and population genomic structure in water mites (Blattner et al., 2022) and ghost‐worms (Cerca et al., 2021). It can also be used to detect copy number variants (Deleye et al., 2017) and most structural variants (Lack et al., 2018). Potential drawbacks are increased cost for WGA kits and GC‐dependent amplification bias (Sabina & Leamon, 2015).

In a previous study, Lack et al. (2018) demonstrated that it is possible to use WGA of *Daphnia pulicaria* resting eggs to achieve DNA concentration suitable for whole‐genome sequencing but caution that it should only be used when necessary, e.g., when it is not possible hatch eggs and sequence genomes of multiple clonal individuals. However, the hatching success of resting eggs is highly species‐dependent, with *Daphnia magna* exhibiting a generally high hatching rate, and members of the *Daphnia longispina* species complex a poor hatching success. Further, within species, other factors such as lake of origin and sediment composition seem to play a role in hatching success (personal observation MC, Radzikowski et al., 2018). In addition, in populations of *D. longispina* species, hybrid resting eggs are less likely to hatch than their parental species in lab experiments (Schwenk et al., 2001) and natural populations (Keller et al., 2007; Keller & Spaak, 2004). Indeed, hybridization and introgression are common in the *D. longispina* species complex. This could result in bias towards the parental species when hatching and rearing clonal lines from resting eggs.

In this study, we tested multiple protocols involving egg isolation, WGA kits and library preparation, and developed a contamination screening to reliably sequence genomes from single resting eggs from the *D. longispina* species complex. We also analyzed the read depth and heterozygosity to optimize this method for future projects involving population genomic investigation of the resting egg bank using recent and historical resting eggs.

## 2 METHODS

### 2.1 Sampling and isolation

Sandy soil was collected by hand from the shoreline of the eutrophic lake Eichbaumsee, Germany (53° 29′ 6″ N, 10° 6′ 11″ E) and stored at 4°C. The exact age of the soil is unknown but the upper layers most likely contain recent *Daphnia* eggs from the last few years.

To collect *Daphnia* eggs small amounts of sediment were sieved (125 μm mesh size) and resuspended in ddH_2_O. Ephippia were eye spotted, counted and transferred to 1.5 ml tubes under a stereo microscope (Nikon SMZ800N). The water was then removed, and the samples were kept at 4°C in the dark until further processing. The ephippia were transferred to a drop of sterile 1x PBS and opened under a stereo microscope with insect needles and forceps previously treated with UV light in a PCR workstation Pro (VWR) and cleaned with DNA‐ExitusPlus (PanReac AppliChem). If an egg was present a picture was taken and the quality was evaluated by eye based on their color into the categories light green or dark green which is the highest quality we find. Eggs that had an already damaged egg membrane, an uneven shape or were orange were discarded (Marková et al., 2006). The resting egg separated from the ephippial casing was then transferred to a tube with sterile 1x PBS with a pipette to wash away the remaining material and the egg was transferred in 1 μl PBS to a new tube with 2, 3 or 14 μl fresh PBS, depending on the WGA protocol (REPLI‐g Mini, Single cell and Single Cell increased sample volume, respectively). The isolated eggs were kept at −20 or −80°C at least overnight until amplification.

### 2.2 DNA extraction from batch cultures

As an unamplified control for the WGA samples, high‐molecular‐weight genomic DNA was extracted from 20 pooled adult *Daphnia* individuals (M5 clone; Nickel et al., 2021) using a modified CTAB extraction method as described in Cristescu et al. (2006).

### 2.3 Whole‐genome amplification of isolated resting eggs

For whole‐genome amplification of single eggs, the REPLI‐g Single Cell Kit and REPLI‐g Mini Kit (Qiagen) were used. Both kits are used for unbiased amplification of genomic loci due to MDA. The REPLI‐g Single Cell Kit can be used for samples of 1–1000 intact cells and yields more DNA. The isolated resting eggs were thawed on ice and WGA was performed following the manufacturer's protocols. Briefly, denaturation buffer was added to the prepared resting eggs in PBS and amplified by phi29 DNA polymerase under isothermal conditions at 30°C for 8 h using the REPLI‐g Single Cell Kit and 16 h using the REPLI‐g Mini Kit and the polymerase was inactivated at 65°C for 3 min. In addition, a modified protocol for the REPLI‐g Single Cell Kit as described by Lack et al. (2018) was used; it is optimized for the amplification of 10–100 ng genomic DNA template and uses an increased sample volume (15 μl).

**FIGURE 1:**
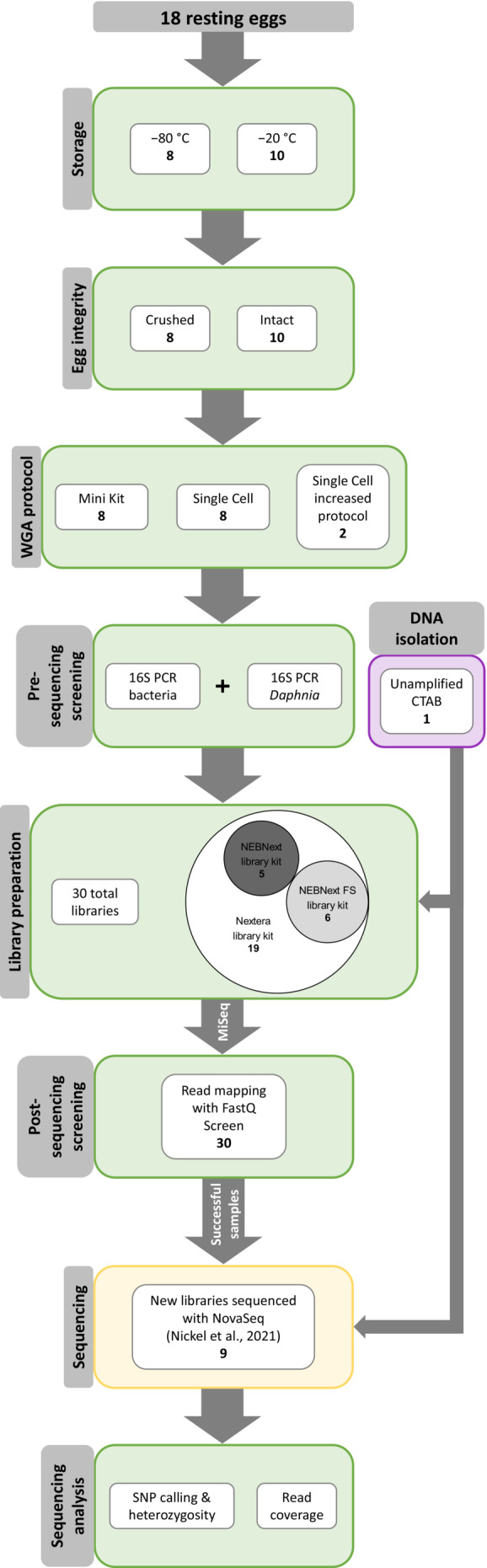
Experimental design workflow for multiple protocols of whole‐genome amplification of isolated resting eggs, library preparation, sequencing, and bioinformatic analysis.

Eggs were either kept intact or punctured with an insect needle before the amplification to test whether manual crushing had an effect. The different methods involving the REPLI‐g kit, if the normal or increased sample volume protocol were used, storage and egg integrity were performed on two eggs each for the nine tested protocol combinations and are shown in Table 1 and Figure 1.

**TABLE 1:** Summary of all samples for egg quality, the complete method used for each egg, and PCR screening results (16S *Daphnia*/16S bacteria: Amplified product visible on a gel).

Sample name	Egg quality	REPLI‐g kit	Protocol	Storage	Egg integrity	16S *Daphnia*	16S bacteria	Total raw reads	Total trimmed reads	Mapped reads %
EIC_18	Dark	Single Cell	Normal	−20°C	intact	Yes	No	Nextera: 106,686	72,564	**93.64**
EIC_15.4	Light	Single Cell	Normal	−20°C	intact	Yes	Yes	Nextera: 55,146	36,468	11.53
EIC_2.1	Unknown	Single Cell	Increased sample	−20°C	intact	Yes	Weak	Nextera: 84,848	54,265	17.91
EIC_2.2	Unknown	Single Cell	Increased sample	−20°C	intact	Yes	Weak	Nextera: 64,268	41,923	20.58
EIC_12.3	Light	Single Cell	Normal	−80°C	intact	Weak	Weak	Nextera: 116,572	78,875	0.04
								NEB: 20,133	19,364	3.64
EIC_13.3	Dark	Single Cell	Normal	−80°C	intact	Yes	No	Nextera: 53,342	35,721	12.12
								NEB: 30,512	29,182	4.29
EIC_3.1	Light	Single Cell	Normal	−20°C	crushed	Yes	Weak	Nextera: 55,176	37,877	8.16
EIC_3.2	Dark	Single Cell	Normal	−20°C	crushed	No	Weak	Nextera: 43,025	26,622	13.82
EIC_16	Light	Single Cell	Normal	−80°C	crushed	Yes	No	Nextera: 156,382	99,335	**95.95**
								NEB FS: 62,699	49,802	**96.12**
EIC_17	Dark	Single Cell	Normal	−80°C	crushed	Yes	No	Nextera: 27,744	17,700	**96.69**
								NEB FS: 63,985	55,836	**96.25**
EIC_7.1	Dark	Mini	Normal	−20°C	intact	Yes	Weak	Nextera: 81,411	53,037	29.99
EIC_7.2	Dark	Mini	Normal	−20°C	intact	No	Yes	Nextera: 42,070	26,465	11.37
								NEB: 40,303	38,387	0.02
EIC_11	Dark	Mini	Normal	−80°C	intact	Yes	Weak	Nextera: 18,787	11,842	**98.06**
								NEB: 43,710	41,774	**96.74**
EIC_12	Light	Mini	Normal	−80°C	intact	Yes	No	Nextera: 63,732	38,445	**97.67**
								NEB: 40,089	38,354	**96.64**
EIC_13	Light	Mini	Normal	−20°C	crushed	Yes	No	Nextera: 102,521	64,463	**97.92**
EIC_13.2	Dark	Mini	Normal	−20°C	crushed	Yes	No	Nextera: 33	9	/
								NEB FS: 63,285	56,212	**97.99**
EIC_14	Light	Mini	Normal	−80°C	crushed	Yes	No	Nextera: 151,989	97,438	**98.23**
								NEB FS: 56,431	49,438	**97.64**
EIC_15	Light, slight dissolving	Mini	Normal	−80°C	crushed	Yes	Weak	Nextera: 89,600	57,955	**97.73**
								NEB FS: 47,563	41,308	**93.89**
M5	/	/	Unamplified CTAB	/	/	Yes	Weak	Nextera: 62,905	37,408	**92.45**
								NEB FS: 104,196	53,906	**93.89**

*Note*: For the sequencing statistics, the number of reads is presented separately for Illumina Nextera or NEB library preparations. The mapped reads % column shows the % of reads mapped to the *D. galeata* genome by BWA. Samples with very high mapping success are considered as successful amplification of *Daphnia* DNA and are indicated in bold while the remaining samples are considered contaminated.

The amplified product was quantified on a NanoDrop spectrophotometer (ThermoFisher) to check that the A260/280 and A260/230 values were both >1.8 which indicates DNA purity. The concentration was measured with a Qubit Fluorometer (ThermoFisher) because during the REPLI‐g reaction single‐stranded DNA is generated by random extension of primer dimers which leads to an overestimation of DNA using a spectrophotometer. Successful amplification product was purified with 0.4x Agencourt AMPure XP magnetic beads (Beckman Coulter) to remove small fragments and eluted in 60 μl 1x TE buffer. The cleaned genomic DNA was then quantified with a Qubit Fluorometer and fragment length was examined on a 4200 TapeStation (Agilent) or Fragment Analyzer (Agilent). The amplification product was stored at −20°C until library preparation. The presence of *Daphnia* DNA in the WGA product was checked by amplifying fragments of the mitochondrial 16S rDNA gene using the universal cladoceran primers S1 (5′‐CGG CCG CCT GTT TAT CAA AAA CAT‐3′) and S2 (5′‐GGA GCT CCG GTT TGA ACT CAG ATC‐3′) with 1 cycle of 93°C for 2 min 30 s, 55°C for 1 min and 72°C for 2 min followed by 40 cycles of 93°C for 1 min, 55°C for 1 min and 72°C for 2 min and running a 1.5% agarose gel at 100 V to assess bands (Schwenk et al., 1998). To check for a low presence of bacterial DNA universal primers for the bacterial 16S rDNA gene were used (5′‐TCC TAC GGG AGG CAG CAG T‐3′ and 5′‐GGA CTA CCA GGG TAT CTA ATC CTG TT‐3′) with 1 cycle of 50°C for 2 min and 95°C for 10 min followed by 40 cycles of 95°C for 15 s and 60°C for 1 min (Nadkarni et al., 2002).

### 2.4 Library preparation and sequencing

Paired‐end library construction was conducted for the 18 WGA samples and one unamplified CTAB sample with the Nextera XT DNA Library Preparation Kit (Illumina). Two library preparation kits were used on the same WGA samples to test which library kit could be best adapted to these samples. Five WGA samples (500 ng as input DNA) were fragmented using the M220 Focused‐ultrasonicator (Covaris) and prepared with the NEBNext® Ultra™ II DNA Library Prep Kit for Illumina® (New England Biolabs). In addition, five WGA samples (500 ng as input DNA) and one unamplified CTAB sample were prepared with NEBNext® Ultra™ II FS DNA Library Prep Kit for Illumina® which includes an enzyme DNA fragmentation step. The obtained fragment length was measured prior to sequencing on a 4200 TapeStation (Agilent) with the High Sensitivity D5000 kit. The libraries were separately pooled and 150 bp paired‐end reads were sequenced for the 19 Nextera libraries and 11 NEB libraries (30 total) on the Illumina MiSeq platform using the MiSeq Reagent Kit v2 Nano (Illumina).

Using only the eight WGA samples that were identified as largely *Daphnia* sequences in the previous low‐coverage MiSeq sequencing step as well as one unamplified CTAB sample (9 total), new libraries were prepared with the NEBNext® Ultra™ II FS DNA Library Prep Kit for Illumina®. Then, 150 bp paired‐end sequencing was generated on the Illumina NovaSeq 6000 platform as part of a previous study (Nickel et al., 2021). This whole‐genome data was used here to have sufficient coverage to assess genome coverage and is available from the European Nucleotide Archive (accession numbers: ERR4610186‐ERR4610192, ERR4610229, and ERR5235052). The successful sample EIC_13.2 could not be sequenced again because no amplification product was left.

### 2.5 Sequencing analysis and genotyping

The quality of raw and trimmed reads was assessed using FastQC v0.11.7 (Andrews, 2010). Trimming and quality filtering of the 30 total MiSeq datasets was performed using Trimmomatic v0.38 (Bolger et al., 2014) with the following parameters: TRAILING: 15 SLIDINGWINDOW: 4:15 MINLEN: 120. To assess contamination in the WGA samples FastQ Screen v0.14.0 with the BWA mapping option was used (Wingett & Andrews, 2018). A custom database was built to map trimmed reads against possible contaminants that included general common contaminants such as *Homo sapiens* (GRCh38.p7), the UniVec database, a bacterial and a viral NCBI reference set all downloaded in April 2018 as well as the *D. galeata* genome (Nickel et al., 2021), *D. magna* genome and *Acutodesmus obliquus* draft genome (Starkenburg et al., 2017; Table A1). All sequences that did not map to any reference genome were filtered and searched against the GenBank nucleotide database (downloaded Feb. 2022) using blastn 2.12.0+ to identify their origin (Camacho et al., 2009). The trimmed reads were mapped to the *D. galeata* reference genome, using BWA v0.7.17 with the mem option (Li & Durbin, 2009) and Qualimap v2.2.1 was used to examine the mapping quality (Okonechnikov et al., 2016).

In addition, adapter trimming and quality filtering of the nine NovaSeq datasets from Nickel et al. (2021) were performed using Trimmomatic v0.38 with the following parameters: ILLUMINACLIP: TruSeq3‐PE.fa:2:30:10 TRAILING: 15 SLIDINGWINDOW: 4:15 MINLEN:70. Following GATK4 best practices for pre‐processing and variant calling (Van der Auwera et al., 2013) the trimmed reads were mapped to the *D. galeata* genome using BWA v0.7.17 with the mem and –M options (Li & Durbin, 2009). Duplicates were marked and filtered out in the BAM file using Picard v2.21.1 (http://broadinstitute.github.io/picard/).

We estimated read depth across the genome with a bin size of 10 kb and normalized with the Reads Per Kilobase per Million mapped reads (RPKM) model using bamCoverage from the deepTools package v3.5.1 (Ramírez et al., 2016).

To call variants for each sample HaplotypeCaller implemented in GATK v4.2.2.0 was run with the —emitRefConfidence GVCF and —include‐non‐variant‐sites option (Poplin et al., 2018). This outputs gVCF files with information on all variant as well as invariant genotyped sites to be able to calculate the total number of genotyped sites within a genomic window. The VCF file was hard filtered to remove variants with a QualByDepth <10, StrandOddsRatio >3, FisherStrand >60, mapping quality <40, MappingQualityRankSumTest <−8, and ReadPosRankSumTest <−5 and indels and multi‐allelic sites were removed with BCFtools v1.9 (Li, 2011). The proportion of heterozygous sites of all genotyped sites was calculated using the Python script popgenWindows.py (github.com/simonhmartin/genomics_general release 0.3) with a sliding 250 kb window and a step size of 25 kb.

## 3 RESULTS

### 3.1 Whole‐genome amplification

The amplification step of genomes derived from resting eggs yielded 3.7–10.4 μg and 9.4–41 μg DNA per reaction for the REPLI‐g Mini and REPLI‐g Single Cell Kit, respectively, and generated very long fragments (fragment length peak 10,000–45,000 bp). Nineteen libraries prepared with Illumina Nextera and eleven libraries prepared with NEB were sequenced on the Illumina MiSeq platform, producing a total of 1,376,237 and 572,906 raw reads, respectively, with an average of 72,434 and 52,082 reads generated per library (Table 1). On average, 62.4% and 87.3% of the reads were retained after trimming and quality control.

### 3.2 Contamination and read mapping of MiSeq datasets

**FIGURE 2:**
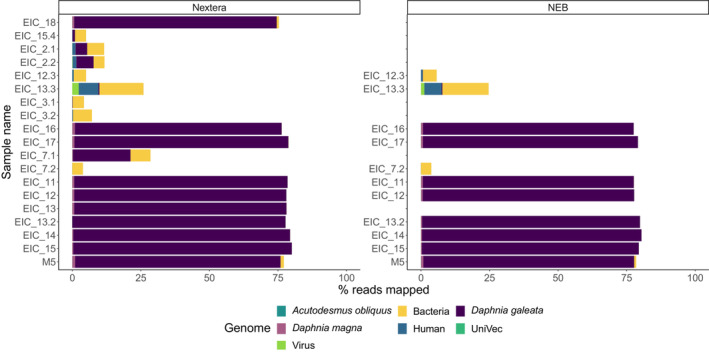
Proportion of reads mapped to a reference genome panel with FastQ Screen for all Illumina Nextera and NEB libraries. Reads that mapped to multiple genomes are not shown.

The trimmed reads were analyzed with FastQ Screen to assess possible contamination. We expected that for samples with successful whole‐genome amplification the majority of reads would map to *D. galeata* with little contamination from other genomes and show similar patterns to the unamplified sample M5. Ten samples indicated the presence of *Daphnia* DNA and mapped >75% to *D. galeata*, <1% to other organisms, and the remaining reads were unmapped or mapped to multiple genomes (Figure 2). However, five samples showed little to no reads mapped to the *D. galeata* genome (0–1%), contamination (~5%) with bacteria, and the remaining reads could not be mapped to a genome included in the panel (Figure 2). For three samples low amounts of reads mapped to the *D. galeata* genome (4–21%) and bacteria (~5%) and the sample EIC_13.3 showed significant human, bacterial and viral contamination most likely caused by contamination during lab work while handling the egg or whole‐genome amplification. Additional BLAST searches of the large percentage of the reads that could not be mapped to any included reference genome in the potentially contaminated samples also revealed no substantial matches to other organisms.

To properly analyze reads that mapped to multiple genomes with FastQ Screen and verify these results, the sequences were mapped separately to the *D. galeata* genome. We clearly identified *D. longispina* species with 92.5%–98.2% of reads from the successful ten samples mapping to the genome and average genome coverage of 0.1x (Table 1). For samples that were already identified by FastQ Screen as having no or small amounts of *D. galeata* reads only 0–30% of reads could be mapped. Samples, where both Nextera and NEB libraries were prepared, gave similar mapping rates and results are presented for both protocols in Figure 2. In total, nine *Daphnia* genomes from resting eggs were successfully amplified and sequenced which results in a 50% success rate as well as one *Daphnia* genome from a pooled unamplified DNA sample.

### 3.3 Effect of different protocols used

Out of the nine failed samples, only two were amplified using the REPLI‐g Mini Kit while seven were amplified using the REPLI‐g Single Cell Kit (normal and increased sample protocol). The latter yielded more DNA but produced lower quality WGA product and we were only able to successfully amplify samples using two protocol combinations (−20°C storage and leaving the eggs intact or −80°C storage and crushing the eggs). While separating resting eggs from the ephippial casing, only high‐quality eggs that showed no sign of degeneration were selected for amplification and classified based on color but there does not seem to be a direct link between higher‐quality dark green eggs and more amplification success. Out of the nine failed samples using either the REPLI‐g Mini or Single Cell Kit, seven were stored at −20°C instead of −80°C.

Three eggs could be successfully amplified after keeping them intact before amplification and six that were manually crushed with an insect needle. However, because of their fragility, the membrane of some of the intact eggs was most likely also punctured during isolation and transfer.

Three eggs out of 10 tested eggs were successfully amplified using the REPLI‐g Single Cell Kit, one using −20°C storage and leaving the eggs intact and two using −80°C storage and crushing the eggs. Six out of 8 tested eggs could be amplified using the REPLI‐g Mini Kit, two each using −80°C storage and either leaving the eggs intact or crushing them and two using −20°C storage and leaving the eggs intact.

The different library kits used generated a similar number of reads per library and a higher proportion of reads were retained after trimming using the NEB kits. In addition, the NEB kit yielded more consistent library concentrations and no failed libraries due to the protocol having more options to customize for different DNA input concentrations.

### 3.4 PCR contamination screening

Two different 16S PCR markers were used to assess the quality of the WGA product before sequencing and to compare these results to the results achieved by sequencing and mapping the reads. Sanger sequencing and BLAST search of the *Daphnia* 16S PCR fragments confirmed that all were of *D. galeata* or *D. longispina* mitochondrial origin. It is to be noted that Sanger sequencing was only conducted here for further diagnosis and is not necessary for the contamination screening.

Three samples had no or weak bands on the *Daphnia* 16S PCR gel and consequently failed during the sequencing. However, six samples where 16S was successfully amplified yielded no sequencing results (Table 1). All but one (EIC_13.3) of the samples that were classified as contaminated after sequencing showed a band during bacterial 16S PCR. However, this was also the case for two non‐contaminated WGA samples and the unamplified control in which we would expect low amounts of bacterial DNA from the animals' microbiota. Combining both results and using only samples that pass *Daphnia* PCR and have no amplification of the bacterial DNA for subsequent sequencing could improve the success rate of WGA samples from 50% without any screening to 88% with stringent criteria. When considering more relaxed criteria that include all samples that passed the *Daphnia* PCR and with weak or no amplification of the bacterial DNA the success rate is 67%.

### 3.5 Read depth and coverage of NovaSeq datasets

While the MiSeq‐generated data worked well to identify contamination quickly and at a lower cost, the low genome coverage (average 0.1x) was not sufficient to assess differences between unamplified and amplified samples and different WGA protocols used. To better compare the mapping rate between samples, assess the read coverage and variant calling, we thus used the sequences of eight successful amplification samples and the unamplified, pooled sample with a higher coverage (0.60–57.05x, Table A2) obtained in another study (Nickel et al., 2021).

As MDA‐WGA can lead to non‐uniform amplification of the genome (Pinard et al., 2006) that could affect variant calling, we checked mapping success and distribution of read coverage across the genome. The mapping rate for all amplified samples was >90% which was similar to those calculated for the MiSeq datasets and the highest rates were achieved for the two samples using the REPLI‐g Mini Kit, −80°C storage, and crushing the eggs (97.76% & 98.26%), the one sample using REPLI‐g Mini Kit, −20°C storage and leaving the eggs intact (97.76%) and one of the samples using REPLI‐g Mini Kit, −80°C storage and leaving the eggs intact (98.06%). The read coverage ranged from 5.55x to 12.41x for the amplified samples except for EIC_12 which showed extremely high adapter content. These were discarded during trimming and it subsequently resulted in lower coverage than the other samples (0.6x).

**FIGURE 3:**
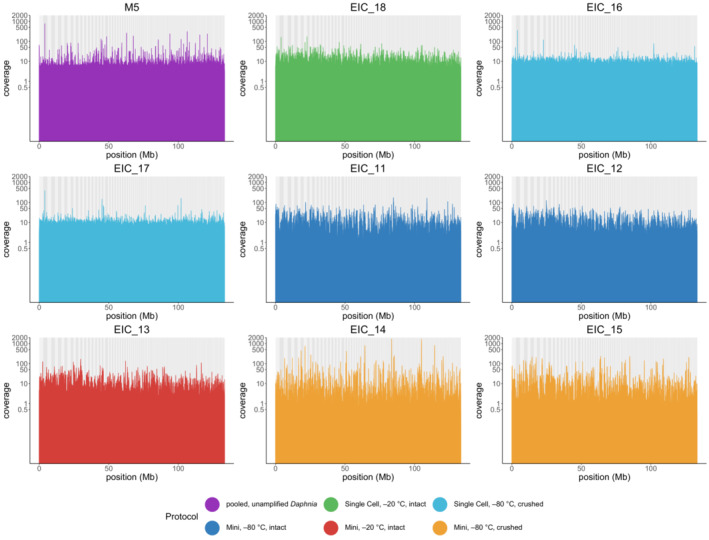
Normalized read depth across the genome in 10 kb bins for each sample. Scaffold positions are marked by alternating gray background bars. Coverage is shown on a log2 scale.

The normalized read depth for the unamplified sample M5 shows uniform coverage across the genome with few regions having low or very high coverage (Figure 3). In general, two samples that were prepared with the same protocol show very similar patterns of read coverage. The three samples that were prepared with the REPLI‐g Single Cell Kit show a uniform coverage similar to the unamplified M5 sample, with most regions having a coverage above 5 and few regions with coverage above 50 that could point to overamplification of specific regions. For the samples that were amplified with the REPLI‐g Mini Kit, most regions have coverage above 1 but some regions show much lower or higher coverage, especially sample EIC_14.

### 3.6 Variant calling and heterozygosity

**FIGURE 4:**
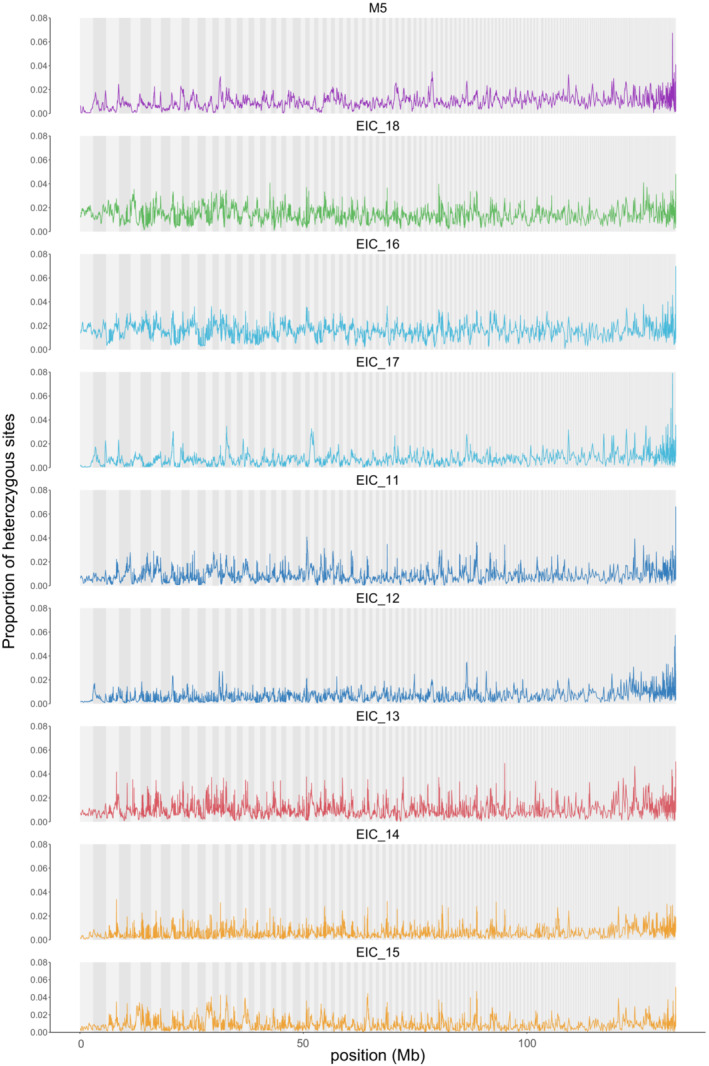
The proportion of heterozygous genotype calls in 250 kb sliding windows across the genome. Scaffold positions are marked by alternating gray background bars. Sample M5 is unamplified and the remaining eight are WGA samples using the same color code for the specific protocol as in Figure 3.

The invariant data set included 130,950,194 sites across the 9 samples. To assess whether we find downwardly biased estimates of heterozygosity in the amplified samples we compared the proportion of heterozygous genotype calls in sliding windows across the genome to the unamplified sample M5 (Figure 4). The heterozygosity across the genome in the unamplified sample was even with few outlier windows and the genome‐wide average heterozygosity was 0.00934. We find very similar genome‐wide patterns for the eight amplified samples and no general trend of loss of heterozygosity, with three samples having higher average heterozygosity compared with M5 and five having lower heterozygosity (Table A3). However, as the resting eggs were sampled from a natural population some differences in heterozygosity between two samples using the same protocol are expected.

## 4 DISCUSSION

Egg banks of zooplankton allow researchers to track long‐term genetic and ecological variation within an ecosystem and provide insight into past populations by hatching long‐dormant eggs or using genetic markers (Alric et al., 2016; Burge et al., 2018; Frisch et al., 2014). However, these studies are still limited by the reduction in egg viability with sediment age and the low amount of high‐quality DNA in the eggs. The goal of this study was to optimize the whole‐genome sequencing of *D. longispina* species resting eggs and establish a more reliable WGA method using 18 single resting eggs isolated from sediment. Hatching eggs from the resting egg bank and establishing clonal lines in the lab can be unpredictable and success rates depend on the species, the water bodies where sediment was collected, and the hatching conditions used (Radzikowski et al., 2018). In addition, *D. longispina* species hybrid resting eggs show lower hatching success and survival rates than their parental species (Schwenk et al., 2001). This introduces bias where hybrids appear less frequent than they are when working on admixed populations from the resting egg bank. We suggest that our method could help get more accurate genomic data from hybrid populations in other studies (Nickel et al., 2021).

In our study, we included the WGA method presented in Lack et al. (2018) using *Daphnia pulicaria* resting eggs where they were able to sequence one out of three resting eggs. We did not achieve successful amplification for the two tested *D. galeata* eggs using the same protocol (EIC_2.1 and EIC_2.2). In addition, Lack et al. (2018) did not use any qualitative diagnostic to assess the amplified *Daphnia* DNA from a resting egg before performing costly library preparation and sequencing steps. The success rate achieved by O'Grady et al. (2021) was much higher for eggs of *Daphnia magna* and *Daphnia pulicaria* (86% and 78%, respectively). Their study also did not include any screening step and so far, no species of the species predominant in European lakes were tested.

Here, different protocols for egg treatment and different library kits were used to successfully amplify and sequence nine genomes from *Daphnia* resting eggs that could be identified as belonging to the *D. longispina* species complex. *Daphnia* and bacterial markers were established to quickly check possible contamination of the WGA product prior to sequencing and exclude contaminated samples before the sequencing step at a low cost, thus improving the success rate to 88%. This may be of particular importance because the success rate can vary across different lakes and may be very low in specific lakes because of the poor condition of resting eggs (Marková et al., 2006). The contamination screening helps to identify potentially successful amplification of *Daphnia* DNA before the library preparation and sequencing and generate genomic data under these difficult conditions. The bacterial markers can be used to identify bacterial contamination in WGA of all other species, while the *Daphnia* markers work for all Cladocera species, many of which also produce resting stages (Vandekerkhove et al., 2005). Our method could be improved with the use of real‐time PCR to directly quantify *Daphnia* DNA in the sample instead of only examining whether it is detectable or not, albeit increasing the cost per sample.

To check for possible amplification bias, genome coverage and heterozygosity from amplified samples were compared with an unamplified DNA sample from pooled adult *Daphnia*. The read mapping ratio was high for all amplified samples and the genome coverage is relatively even with few outlier regions and very similar patterns for samples using the same protocol. Loss of heterozygosity that could impact genotyping was not observed in all samples. While the REPLI‐g Single Cell Kit generally achieves good results for those metrics the low reproducibility of the amplification makes it unsuitable for our purpose.

Some challenges with whole‐genome amplification were contamination which most likely stemmed from problems during the amplification step. Lab protocols to minimize contamination were used but the high sensitivity of the WGA kits could lead to the amplification of DNA from the wrong cells present in the sample.

A critical step is to use high‐quality undamaged eggs that show no signs of degradation. In eggs from older sediment layers, this is often more difficult and will be tested in a different study. The benefits of this method included the potential to go back decades to centuries because DNA is preserved longer than eggs that can be reliably hatched (Limburg & Weider, 2002). However, some amplifications of resting eggs failed but we were not able to identify one or multiple specific organisms as major contaminants and most reads could not be mapped. Instead, we hypothesize that these unknown sequences could be caused by the phi29 polymerase performing non‐templated DNA synthesis which is a known phenomenon in MDA‐WGA and produces “junk” DNA possibly when the egg is already degrading or the amount of DNA present is too small (Nelson, 2014). This seems to be a more frequent problem using the REPLI‐g Single Cell Kit where only three out of ten samples were successfully amplified.

In conclusion, the most appropriate complete protocol we tested included using the REPLI‐g Mini Kit, storing eggs at −80°C, leaving the eggs intact, and using the NEB library kit. After the resting egg is removed from the ephippia, it is extremely fragile, and immediately freezing it at −80°C seems to be a crucial step to slow DNA degradation. Nevertheless, the high biological variability of the resting eggs and the relatively small number of eggs tested for each protocol makes it difficult to draw more general conclusions.

The possible shortcomings of WGA methods include the amplification of contaminant DNA instead or in addition to the template DNA (Thoendel et al., 2017), and increased costs for sample preparation. Currently, the cost for the suggested REPLI‐g Mini Kit is ~$8 per sample. The amplification of contaminant DNA remains an issue; however, our contamination screening when applied at a larger scale would lead to substantial cost savings, by markedly reducing the number of contaminated samples being processed further and sequenced. While sequencing itself has become extremely low‐cost, library preparation remains costly. Prices for reagents, kits, labor, and sequencing services vary considerably between countries and are further influenced by the scale of purchasing. We, therefore, refrain from providing exact cost calculations. When using WGA strategies, it is also important to consider the impact of the quantity of input DNA that can lead to downwardly biased estimates of heterozygosity and therefore genotyping bias (Medeiros & de Medeiros & Farrell, 2018). This study and others that use very small amounts of input DNA (Campbell et al., 2020; Cruaud et al., 2018; O'Grady et al., 2021) indicate that MDA‐WGA does not introduce amplification bias that affects SNP genotyping. It is also suitable for structural variant calling with the exception of inversions (Lack et al., 2018).

To sum it up, our method will allow the resequencing of resting eggs from different sediment layers to characterize genomic changes through time in the *D. longispina* species complex. In a broader context, WGA could also be used for resting stages of other organisms with low amounts of DNA such as other Cladocera taxa, rotifers, or diatoms to gain a more complete understanding of freshwater ecosystems.

## AUTHOR CONTRIBUTIONS

**Jana Nickel:** Conceptualization (equal); data curation (equal); formal analysis (equal); investigation (lead); methodology (equal); visualization (equal); writing – original draft (equal); writing – review and editing (equal). **Mathilde Cordellier:** Conceptualization (equal); funding acquisition (equal); investigation (supporting); methodology (equal); resources (equal); supervision (equal); writing – review and editing (equal).

## CONFLICT OF INTEREST

The authors declare that they have no conflict of interest.

## Data Availability

The NovaSeq short read data is available in the European Nucleotide Archive under run accession numbers ERR4610186‐ERR4610192, ERR4610229, and ERR5235052 and the MiSeq datasets are deposited in Zenodo (https://doi.org/10.5281/zenodo.7253120).

## APPENDIX A

**TABLE A1:** Accession number for genomes included in the custom database to assess contamination with FastQ Screen.

Genome	GenBank accession
*Homo sapiens*	GCA_000001405.22
UniVec	https://ftp.ncbi.nlm.nih.gov/pub/UniVec/
Bacteria	https://ftp.ncbi.nlm.nih.gov/refseq/release/bacteria/
Virus	https://ftp.ncbi.nlm.nih.gov/refseq/release/viral/
*Daphnia galeata*	GCA_918697745.1
*Daphnia magna*	GCA_001632505.1
*Acutodesmus obliquus*	GCA_002149895.1

**TABLE A2:** Summary of sequencing statistics for all successful amplification samples that were sequenced to a higher coverage using NovaSeq.

Sample name	Total raw reads	Total trimmed reads	Mapped reads %	Mean genome coverage
EIC_18	14,952,714	7,897,119	94.61	7.32
EIC_16	15,450,664	6,755,689	96.55	6.52
EIC_17	15,392,966	11,433,190	96.67	11.43
EIC_11	16,210,646	5,563,028	98.06	5.55
EIC_12	19,121,834	929,386	90.95	0.60
EIC_13	14,463,272	11,446,924	97.76	11.55
EIC_14	16,917,644	10,256,933	98.26	10.39
EIC_15	15,531,160	12,295,702	97.76	12.41
M5	77,594,732	65,570,833	84.75	57.05

*Note*: Mean genome coverage is based on the estimated genome size of 156 Mb for *D. galeata*.

**TABLE A3:** Average genome‐wide heterozygosity estimated from the proportion of heterozygous genotype calls in sliding windows across the genome.

Sample name	Average genome‐wide heterozygosity
EIC_18	0.01100
EIC_16	0.01225
EIC_17	0.00613
EIC_11	0.00697
EIC_12	0.00633
EIC_13	0.00980
EIC_14	0.00598
EIC_15	0.00756
M5	0.00934
